# Prediction of Contact Fatigue Performance Degradation Trends Based on Multi-Domain Features and Temporal Convolutional Networks

**DOI:** 10.3390/e25091316

**Published:** 2023-09-09

**Authors:** Yu Liu, Yuanbo Liu, Yan Yang

**Affiliations:** College of Mechanical Engineering, Chongqing University of Technology, Chongqing 400054, China; liuyu@cqut.edu.cn (Y.L.); yuanboliu0618@163.com (Y.L.)

**Keywords:** contact fatigue, feature extraction, health indexes (HIs), degradation prediction, temporal convolutional network (TCN), convolutional autoencoder (CAE)

## Abstract

Contact fatigue is one of the most common failure forms of typical basic components such as bearings and gears. Accurate prediction of contact fatigue performance degradation trends in components is conducive to the scientific formulation of maintenance strategies and health management of equipment, which is of great significance for industrial production. In this paper, to realize the performance degradation trend prediction accurately, a prediction method based on multi-domain features and temporal convolutional networks (TCNs) was proposed. Firstly, a multi-domain and high-dimensional feature set of vibration signals was constructed, and performance degradation indexes with good sensitivity and strong trends were initially screened using comprehensive evaluation indexes. Secondly, the kernel principal component analysis (KPCA) method was used to eliminate redundant information among multi-domain features and construct health indexes (HIs) based on a convolutional autoencoder (CAE) network. Then, the performance degradation trend prediction model based on TCN was constructed, and the degradation trend prediction for the monitored object was realized using direct multi-step prediction. On this basis, the effectiveness of the proposed method was verified using a bearing common-use data set, and it was successfully applied to performance degradation trend prediction for rolling contact fatigue specimens. The results show that using KPCA can reduce the feature set from 14 dimensions to 4 dimensions and retain 98.33% of the information in the original preferred feature set. The method of constructing the HI based on CAE is effective, and change processes versus time of the constructed HI can truly reflect the degradation process of rolling contact fatigue specimen performance; this method has obvious advantages over the two commonly used methods for constructing HIs including auto-encoding (AE) networks and gaussian mixture models (GMMs). The model based on TCN can accurately predict the performance degradation of rolling contact fatigue specimens. Compared with prediction models based on long short-term memory (LSTM) networks and gating recurrent units (GRUs), the model based on TCN has better performance and higher prediction accuracy. The RMS error and average absolute error for a prediction step of 3 are 0.0146 and 0.0105, respectively. Overall, the proposed method has universal significance and can be applied to predict the performance degradation trend of other mechanical equipment/parts.

## 1. Introduction

With the rapid progress of manufacturing technology, the structure of mechanical equipment becomes more and more complex, the operating conditions of parts become more and more severe [1], and the probability of failure increases accordingly. Therefore, predictive monitoring of mechanical equipment/parts is particularly important because it helps in the scientific formulation of maintenance strategies and the health management of equipment. At the same time, the application of technologies such as the Internet, smart sensors, and wireless communication in the mechanical field enables more information to be collected. These massive data reflect the health status and performance changes in mechanical equipment/parts and contain rich information, which has prompted the field of mechanical health monitoring to enter the era of “big data”. However, existing fault diagnosis and early warning methods cannot reliably realize fault diagnoses and early warnings for the status of mechanical equipment/parts under the background of massive data. As an alternative, data-driven mechanical equipment/parts health monitoring technology is attracting extensive attention from researchers [2,3]. Therefore, identifying methods to deeply mine and utilize historical and real-time information in massive data to accurately grasp the health status of mechanical equipment/parts and analyze its performance degradation trends has become a research hotspot in the condition monitoring field [4].

The failure process of contact fatigue is essentially the initiation and propagation of cracks. Contact fatigue strength is an important index that affects the service performance of bearings, gears, etc. At present, rolling contact fatigue testing is the only way to obtain the contact fatigue properties of materials, which takes a long time and is labor-intensive. Therefore, it is of great significance to realize accurate predictions for performance degradation trends of rolling contact fatigue specimens. This will not only predict the fatigue failure time point of specimens in advance and shorten the test time but also improve the methods used to obtain contact fatigue properties of materials. That is to say, in the context of big data, the contact fatigue properties of materials can be obtained by analyzing historical data of materials rather than relying only on rolling contact fatigue tests.

To predict the degradation trends of monitored objects, the core work mainly includes two parts. One is to construct an HI that can truly reflect the performance degradation process of the monitored object throughout its life cycle. The other is to build a predictive model that can accurately predict the changing trend in the HI. For the construction of an HI, one idea is to extract features from raw data and use them for state monitoring. This method is widely used, such as extracting single-domain multi-features and multi-domain multi-features, as well as directly extracting deep features from raw data, etc., and directly using the extracted features as the HI or using the dimension-reduced features as the HI [5,6,7,8,9,10,11,12]. For example, Ahmad W et al. [5] extracted time domain features such as the rms, skewness, and mean value of rolling bearing vibration signals for use as an HI. Zhang Y Q et al. [10] extracted three indexes for use as an HI, including the root mean square value, steepness, and average power spectral density of bearings, and realized the early fault identification of rolling bearings. Another method is to divide the feature data into healthy samples and test samples based on the extracted features, measure the health degree of the test samples by calculating the similarity, and then define the health degree as the HI. The change process in the health degree is regarded as the process of performance degradation [13,14,15,16]. For example, Zheng X X et al. [13] first extracted the wavelet packet feature of the vibration signal of the rolling bearing and then used the Markovis distance between the features as the HI of the bearing. Yang C et al. [15] used the grey correlation degree as the HI of bearing performance, which was able to detect bearing faults earlier. In addition, some researchers directly use the raw signal as the input for neural networks [17,18,19,20] and use the deep features contained in the extracted signal as an HI. For example, Yang L et al. [19] reported a potential method to realize real-time monitoring of sediment particle parameters by introducing a particles-laden droplet-driven triboelectric nanogenerator (PLDD-TENG) combined with the deep learning method. Their results indicated that a convolutional neural network (CNN) deep learning model could identify the particle parameters based on the output signals of PLDD-TENG and high identifying accuracy. However, the first two methods have problems such as incomplete use of the state information of the monitored object and ignoring the nonlinear relationship among characteristics. The third method needs to fully consider the operating characteristics of the monitored objects because different objects have different mechanical structures, operating environments, and damage mechanisms. In terms of building degradation trend prediction models, most researchers use shallow machine learning methods, such as support vector machine (SVM) and extreme learning machine (ELM) [21,22,23,24], or the long short-term memory (LSTM) neural network, gating recurrent unit (GRU) model, and deep learning method optimized on this basis [25,26,27,28,29,30,31,32]. However, it is difficult for shallow machine learning methods to fully exploit the correlation between data, which will have a great impact on prediction accuracy. For deep learning methods, the current recurrent neural network model is most suitable for sequence data prediction; however, it is still difficult to perform a large number of parallel calculations due to the network structure, which affects the calculation speed of the model.

**Figure 1:**
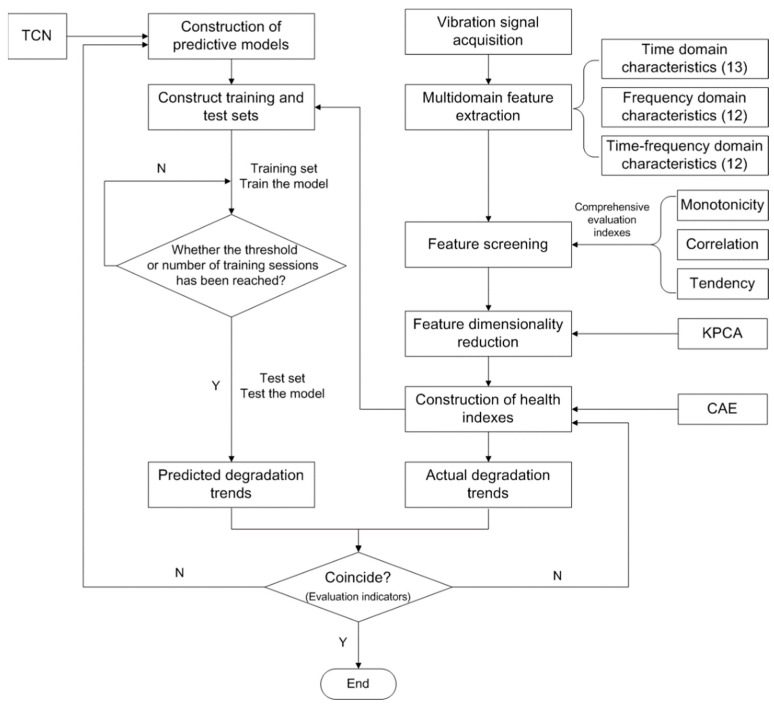
A flowchart outlining the proposed method.

The vibration signal contains the most essential information on the operating state of the monitored object [33]. In this paper, we carry out research on the performance degradation prediction of a rolling contact fatigue specimen based on the vibration signal. A performance degradation trend prediction method based on multi-domain features and TCN is proposed, aimed at addressing the shortcomings of existing performance degradation prediction research, such as information loss when building an HI, poor parallel computing performance, and a small receptive field when building a prediction model, combined with non-stationary and nonlinear vibration characteristics of the monitored object and time series characteristics of monitoring object performance degradation. A flowchart is given in Figure 1, and the effectiveness of the method was verified using a bearing common use data set. On this basis, the proposed method was successfully applied to the performance degradation trend prediction for a rolling contact fatigue specimen.

## 2. Theory and Method

### 2.1. Multi-Domain Feature Extraction and Feature Screening

#### 2.1.1. Multi-Domain Feature Extraction

In view of the fact that the information contained in a single feature or single-domain feature has certain limitations, it cannot fully reflect the complete process of performance degradation for a monitored object. Therefore, this paper extracts the vibration signal from the time domain, frequency domain, and time-frequency domain. Note that the discrete vibration signal value collected each time during the test is $x(n)=\left\{x_{1},x_{2},\cdots x_{N-1},x_{N}\right\}$, where *N* is the number of sampling points for each time. For the convenience of subsequent description, the extracted features are numbered.

Time domain analysis is to describe vibration signals with statistical features, including dimensional indicators and dimensionless indicators. In this paper, seven dimensional features are extracted, such as mean and root mean square value, and six dimensionless features extracted, such as the skewness index and kurtosis index, which are listed in Table 1 [34].

The frequency domain analysis is to transform the vibration signal into the frequency domain using Fourier transform (FT) and then analyze the obtained signal spectrogram. Compared with time domain analysis, the frequency domain analysis can display more details of fault characteristics. In this paper, the 12 frequency domain features that were extracted, such as the frequency amplitude mean and frequency amplitude variance, are shown in Table 2 [34], where *s*(*k*) represents the spectrum obtained using the fast Fourier transform (FFT) of each sampled signal and *f_k_* represents the frequency value of the *k*-th spectral line.

**Figure 2:**
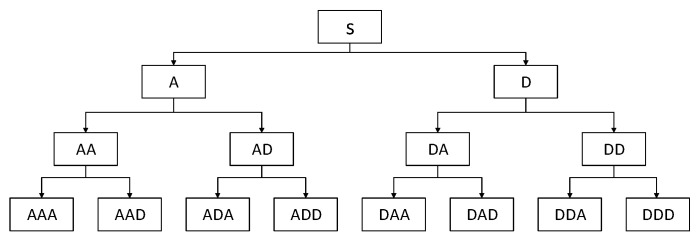
Schematic diagram showing the three-layer wavelet packet decomposition.

Time–frequency analysis can capture transient characteristics, making up for the limitation that a single time–domain analysis or frequency–domain analysis cannot fully reflect fault characteristics when dealing with complex non-stationary signals. In this paper, the wavelet packet transform is used to extract the time–frequency domain features of the vibration signal, and the wavelet basis is used to select the db3 function to decompose the signal in three layers, as shown in Figure 2.

After the original signal is decomposed with the wavelet packet, the second norm of the node coefficient is called the node energy. When the monitored object fails, some node energy may change. Therefore, the ratio of the node energy to the total node energy in the corresponding decomposition layer is used as the time–frequency domain feature parameter.

(1) $$e_{i}={\left\|c{\left(t\right)}_{i}\right\|}_{2}$$

(2) $$E_{i}=e_{i}/\sum_{m=1}^{M}e_{m}$$

where *e_i_* represents the energy of the *i*-th node coefficient; *c*(*t*)*_i_* represents the wavelet packet coefficient of the *i*-th node decomposed at each layer; and *M* represents the total number of nodes obtained with each layer decomposition.

Considering the feature number and the decomposition degree of the signal, this paper selects a total of 12 feature parameters for the energy ratio of each node in the second and third layers of the signal wavelet packet decomposition as the time–frequency domain features. According to the position of each node in the wavelet packet signal decomposition tree diagram shown in Figure 2, for the second layer and the third layer, in order from left to right, the eigenvalues calculated using the coefficients of each node are numbered as *f*_26_–*f*_29_ and *f*_30_–*f*_37_.

#### 2.1.2. Feature Screening

A total of 37 multi-domain features were extracted based on the original vibration signal, but not every feature adequately reflects the performance degradation process of the monitored object. Therefore, it is unscientific to screen features only based on the visualization results of features; instead, it is necessary to establish evaluation indicators to obtain the optimal feature set. In this paper, the monotonicity of features, the correlation between features, and the trend evaluation index of features are integrated [35], and a comprehensive evaluation index *Ch*(*F*) is constructed for feature screening. It should be noted that, since a change in the feature may show a downward trend with time, where its trend value is negative, the trend value in Equation (3) takes an absolute value.

(3) $$\begin{array}{l} Ch(F)=\alpha_{1}Mon(F)+\alpha_{2}\bar{Con(F)}+\alpha_{3}\left|Tre(F)\right|\\ s.t.\begin{array}{cc} & \sum_{i=1}^{3}\alpha_{i}=1 \end{array},\alpha_{i}>0 \end{array}$$

where *a*_1_, *a*_2_, *a*_3_ represent the proportion of monotonicity, correlation mean, and trend in the feature, respectively, and its value can be optimized according to the service environment and performance degradation characteristics of the monitored object. The definitions of three evaluation indicators are shown in Equations (4)–(6), respectively.

(4) $$Mon\left(F\right)=\frac{1}{N-1}\left|S(\frac{d}{dF}>0)-S(\frac{d}{dF}<0)\right|$$

(5) $$\bar{Con(F)}=\frac{1}{N}\sum_{j=1}^{N}\left|Con(F,F_{j})\right|$$

(6) $$Tre(\tilde{F},\tilde{T})=\frac{N\left(\sum_{i=1}^{N}{\tilde{f}}_{i}{\tilde{t}}_{i}\right)-\left(\sum_{i=1}^{N}{\tilde{f}}_{i}\right)\left(\sum_{i=1}^{N}{\tilde{t}}_{i}\right)}{\sqrt{\left[N\sum_{i=1}^{N}{\tilde{f}}_{i}{}^{2}-{\left(\sum_{i=1}^{N}{\tilde{f}}_{i}\right)}^{2}\right]\left[N\sum_{i=1}^{N}{\tilde{t}}_{i}{}^{2}-{\left(\sum_{i=1}^{N}{\tilde{t}}_{i}\right)}^{2}\right]}}$$

In Equation (4), *N* represents the number of samples in the feature *F*; *d*/*dF* represents the derivation result for the feature *F*; *S* represents the number of *d*/*dF* greater than zero or less than zero. In Equation (5), if there are *N* feature sequences, the correlation between the feature and the features including itself can be obtained using Equation (7) (the correlation between the feature and itself is 1), and then the obtained value can be substituted into Equation (5) and used to obtain the mean correlation of features. In Equation (6), ${\tilde{f}}_{i}$ represents the *i*-th value in the rank sequence $\tilde{F}$ of the feature sequence *F* and ${\tilde{t}}_{i}$ represents the *i*-th value in the rank sequence $\tilde{T}$ of the time series *T*.

(7) $$Con(F_{1},F_{2})=\frac{\sum_{i=1}^{N}\left(f_{1,i}-{\bar{F}}_{1}\right)\left(f_{2,i}-{\bar{F}}_{2}\right)}{\sqrt{\sum_{i=1}^{N}{\left(f_{1,i}-{\bar{F}}_{1}\right)}^{2}\sum_{i=1}^{N}{\left(f_{2,i}-{\bar{F}}_{2}\right)}^{2}}}$$

where *F*_1_, *F*_2_ represents any two feature sequences; *N* represents the number of samples in the feature; *f*_1,*i*_ and ${\bar{F}}_{1}$ represents the *i*-th eigenvalue and mean of the feature *F*_1_, respectively; and *f*_2,*i*_ and ${\bar{F}}_{2}$ represent the *i*-th eigenvalue and mean of the feature *F*_2_, respectively.

In order to obtain the features that are sensitive to the performance degradation process of the monitored object, the monotonicity, correlation mean, and trend of 37 multi-domain features are calculated using Equations (4)–(6). On this basis, the comprehensive evaluation index value of each feature is calculated using Equation (3), and the features with larger comprehensive evaluation index values are selected to form the optimal feature set.

### 2.2. Construction of Health Indexes

The construction of HIs is closely related to the extracted vibration signal characteristics, and the ideal HI can clearly reflect the performance degradation process of the monitored object during its whole life cycle. Aiming at the information loss problem faced by the existing one-dimensional HI, on the basis of the above multi-domain feature extraction, this paper uses kernel principal component analysis (KPCA) to reduce the feature dimension and uses a convolutional autoencoder (CAE) network to build an HI.

#### 2.2.1. Feature Dimensionality Reduction Based on KPCA

The dimension of the preferred feature set obtained using the aforementioned method is still high, which will bring dimensional disaster to subsequent calculations. At the same time, there is coupling between the features of the high-dimensional feature set, which will lead to information duplication and overfitting. Therefore, it is necessary to reduce the dimension of the preferred feature set to retain as much effective information as possible while reducing the dimension of the feature set.

Considering the non-stationary and nonlinear characteristics of vibration signals in the process of performance degradation of most monitored objects, it is more general to select a dimension reduction method suitable for nonlinear operations for feature dimension reduction. In this paper, the KPCA method is used to reduce the dimension of the preferred feature set. This method uses a kernel function to effectively capture the nonlinear characteristics of the data [14] and non-linearly maps the linearly inseparable data in low-dimensional space to high-dimensional space, so as to realize the linearity of the data [36,37].

The basic idea of KPCA is to achieve data classification and fitting by transforming data originally located in a low-dimensional feature space into a higher-dimensional feature space. In this method, the original data are mapped into a higher-dimensional space, a new kernel matrix is constructed using the inner product, and then feature value decomposition of the kernel matrix is performed to extract the most important feature vectors. When the original data are projected onto these feature vectors, the original data can be converted into a nonlinear feature space to achieve nonlinear feature extraction [38].

#### 2.2.2. Construction of the HI Based on a CAE Network

**Figure 3:**
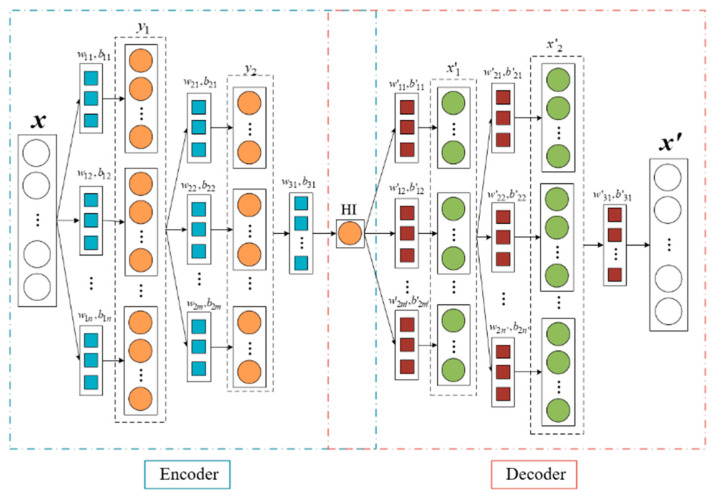
Schematic diagram showing a convolution autoencoder network.

A convolution autoencoder (CAE) network is a network model derived from an autoencoder (AE) network, which converts the encoding and decoding calculation process of data from conventional linear layers to convolution layers to better obtain data deep nonlinear information. The simple CAE network structure is shown in Figure 3.

In the encoding stage, the input information is encoded using multi-layer convolution operations, after which a one-dimensional encoded feature is obtained. For a one-dimensional input sequence *x*, the one-dimensional convolution operation at each layer is as follows:

(8) $$y_{k}=f(w_{k}\cdot x+b_{k})$$

where *w_k_* represents the *k*-th convolution kernel of the convolutional layer; *b_k_* represents the corresponding bias; “·” represents the convolution operation; and *f* represents the activation function. 

In the decoding stage, the encoded features are used as input, and the dimension of the original information is reconstructed using layer-by-layer one-dimensional transposed convolution. The 1D transposed convolution operation for each layer is as follows:

(9) $$x_{k}’=h(w{’}_{k}*y+b{’}_{k})$$

where *y* represents the input sequence of the transposed convolution; $w{’}_{k}$ and $b{’}_{k}$ represent the *k*-th convolution kernel and the corresponding bias of the transposed convolution, respectively; “∗” represents the transposed convolution operation; and *h* represents the activation function.

Compared with the fully connected layer in the ordinary AE network, this paper uses the convolution layer in the CAE network, which has a stronger nonlinear mapping ability. The network architecture and parameters of CAE are shown in Table 3.

### 2.3. Performance Degradation Prediction

The HI constructed above can reflect the degradation process for the performance of the monitored object. Therefore, the prediction for the performance degradation trend of the monitored object is essentially the prediction of the HI value. The HI is one-dimensional time series data that changes with time. Aiming at the shortcomings of the commonly used prediction models based on recurrent neural networks, such as low parallel computation and a small receptive field for input data, this paper builds a performance degradation trend prediction model for a monitored object using a temporal convolutional network (TCN) and realizes the prediction. TCN adopts the structure of one-dimensional full convolution, causal convolution, dilated convolution, and residual connection, which has the advantages of a large receptive field and strong parallel computing and can weaken the gradient problem [39,40].

#### 2.3.1. Temporal Convolutional Networks

**Figure 4:**
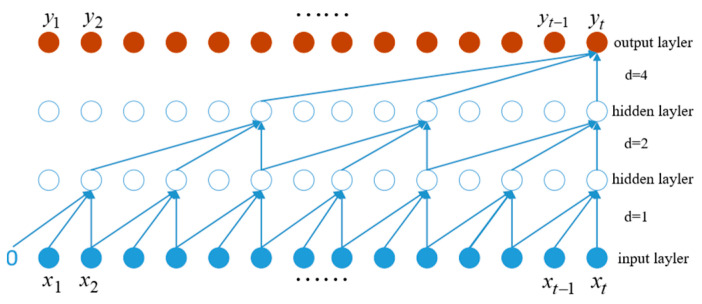
Schematic diagram showing the dilated causal convolution operation.

Figure 4 illustrates the process of representing one-dimensional full convolution, causal convolution, and dilated convolution with dilated causal convolution, where the input sequence is $X=\left\{x_{1},x_{2},\cdots ,x_{t-1},x_{t}\right\}$, the output sequence after the three-layer one-dimensional dilated causal convolution operation with a convolution kernel size of 3 is $Y=\left\{y_{1},y_{2},\cdots ,y_{t-1},y_{t}\right\}$, and the dilation coefficient in the convolution calculation d∈N*, generally *d* = 2.

The receptive field *v* is related to the size of the convolution kernel, the number of layers in the convolution calculation, and the expansion coefficient. Its calculation formula is:

(10) $$v=1+\sum_{i=0}^{l-1}(k-1)\cdot b^{i}$$

where *k* represents the size of the convolution kernel; *l* represents the number of convolution layers in the network; and *b* represents the base of the expansion coefficient, usually set as *b* = 2.

In TCN, given a one-dimensional input sequence $x\in R^{n}$ and a convolution kernel f:{0,1⋯k−1}→R, the calculation result for the dilated causal full convolution at position 3 of the sequence is:

(11) $$F(s)=\sum_{i=0}^{k-1}f(i)\cdot x_{s-d\cdot i}$$

where $x_{s-d\cdot i}$ represents the (s−d⋅i)-th element in the previous layer and the other parameters have the same meanings as before.

**Figure 5:**
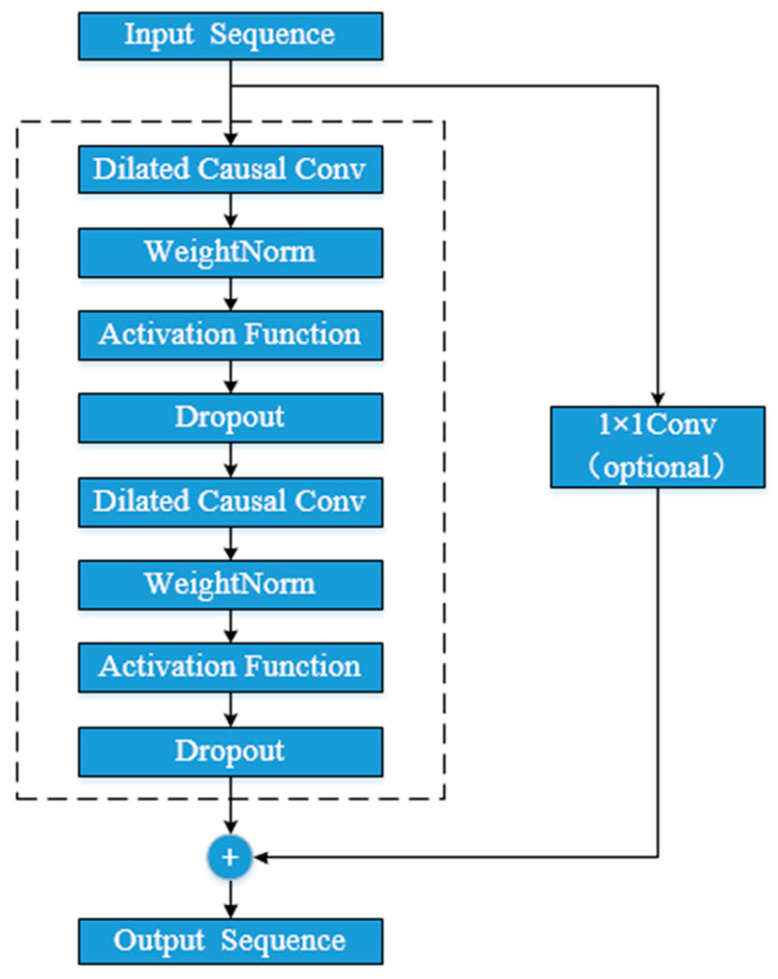
Schematic diagram showing the residual block in TCN.

In addition, TCN uses residual block connection to increase the network depth. By connecting multiple residual blocks together, the gradient problem can be effectively weakened, and the model receptive field can be further increased. The form of each residual block is shown in Figure 5.

In this paper, the input length of the input sample is 8, the expansion coefficient is 2, the convolution kernel size is 4, the number of convolution kernels is 5, the number of convolutional layers is 3, and the prediction step size can be modified to 3, 4, and 5 as needed. 

#### 2.3.2. Performance Degradation Prediction Based on TCN

Data-driven forecasting can be divided into single-step prediction and multi-step prediction. Since single-step prediction can only predict the value of the next moment, it is of little practical engineering significance. Therefore, this paper uses multi-step prediction.

**Figure 6:**
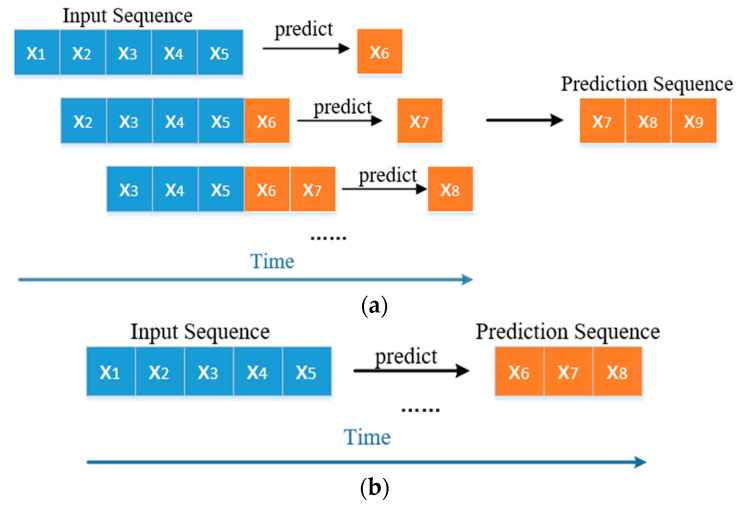
Multi-step forecasting. (**a**) Recursive multi-step prediction. (**b**) Direct multi-step forecasting.

The strategy of multi-step forecasting is divided into direct forecasting and recursive forecasting [4], as shown in Figure 6. There is only one time point for each prediction in recursive prediction. Since the errors in each prediction will accumulate, it may eventually cause a large deviation between the predicted sequence and the actual value. Direct prediction is used to predict the target sequence at one time. Since there are many time points for each prediction, the prediction model must be better. Comparing the two forecasting strategies and considering the engineering application value, this paper uses direct forecasting.

The process of using direct multi-step prediction to predict the performance degradation of the monitoring object is as follows: (1) Combine the HI sample numbers of the monitored objects, divide them into training sets and test sets according to an appropriate ratio, use spatial phase reconstruction technology to form the training set and test set for the prediction model, respectively, and set the time step and prediction step for each input. (2) Set the key parameters of the prediction model and model training, input the training set into the prediction model, use the obtained predicted value and the actual value to calculate the error, and optimize the hyperparameters in the model using error backpropagation. This cycle is repeated until the set number of training times is reached or the prediction error reaches the threshold. (3) Input the test set into the trained model to obtain the predicted value, calculate the evaluation index based on the error between the predicted value and the actual value, and evaluate the prediction ability of the model.

In this paper, RMS error (*RMSE*) and mean absolute error (*MAE*) are used as the evaluation indicators for the model, and their calculation formulas are:

(12) $$RMSE=\sqrt{\frac{1}{N}\sum_{i=1}^{N}{\left(\hat{y}-y\right)}^{2}}$$

(13) $$MAE=\frac{1}{N}\sum_{i=1}^{N}\left|\hat{y}-y\right|$$

where $\hat{y}$ represents the predicted value of the model and *y* represents the true value.

## 3. Verification

**Figure 7:**
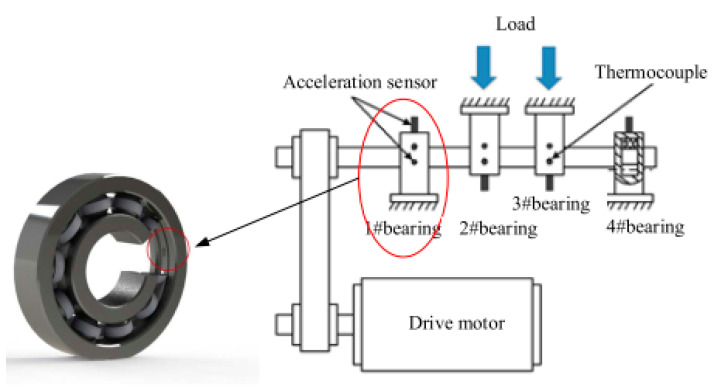
Rolling bearing full life test bench.

The data used in the verification test come from the rolling bearing life data set of the NSF I/UCR Intelligent System Maintenance Center in the United States. The structure of the test bench is shown in Figure 7. The DC motor drives the drive shaft to rotate with a speed 2000 r/min. The applied radial load is 6000 lbs (i.e., 2721.5 kg), and four double-row cylindrical roller bearings (Rexnord ZA-2115) are installed on the drive shaft. The 353B33 high-sensitivity ICP accelerometer from the PCB company and the 6062E acquisition card from the NI company are used to collect the vibration signal of the bearing.

The bearing public data contain three test data, and the second test data are used in this paper. Among them, the sampling frequency of the experiment is 20 kHz, data are collected every 10 min, and 20,480 sampling points are collected each time. The test ran for a total of 8 days. The acceleration signals of the four bearings corresponded to the four channels in the data set, respectively. Finally, the test ended when the outer ring of the 1#bearing failed. A total of 984 times were collected. Therefore, a total of 984 × 20,480 sampling points were collected. 

**Figure 8:**
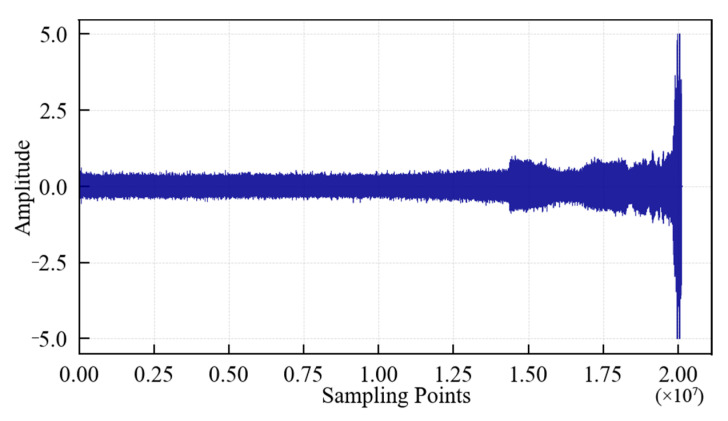
Lifetime vibration signal of the 1#bearing.

The change in the vibration signal during the whole life of the 1#bearing is shown in Figure 8. It can be seen that: (1) In the early stage of the test, the running state of the 1#bearing was relatively stable, and its vibration signal had no obvious abnormal change. (2) After a minor fault occurs in the 1#bearing, the amplitude of the vibration signal begins to increase. (3) With the progress of the test, the failure of the 1#bearing intensified, the vibration signal began to change abruptly, the amplitude further increased, and finally, the 1#bearing suffered a serious outer ring failure. The value of the vibration signal at the corresponding time also reflected this situation more clearly. The above phenomenon shows that it is feasible to analyze the bearing performance degradation trend using the vibration signal. However, the degradation trend in the original vibration signal is not intuitive enough to analyze the performance degradation process of the 1#bearing. The 1#bearing fault will only be reflected in the original vibration signal when it is serious, which is obviously disadvantageous for the identification of early/weak faults. Therefore, it is necessary to analyze the performance degradation process of the 1#bearing using feature extraction.

### 3.1. Verification of the Feature Screening Method

**Figure 9:**
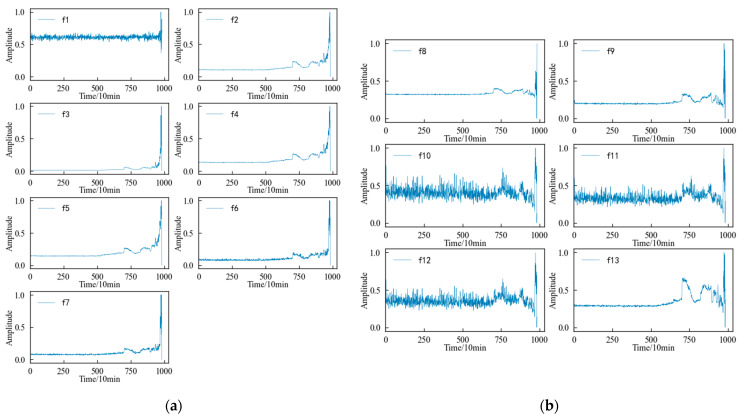
Variation trend in time domain feature of 1#bearing. (**a**) Dimensional time domain features. (**b**) Dimensionless time domain features.

**Figure 10:**
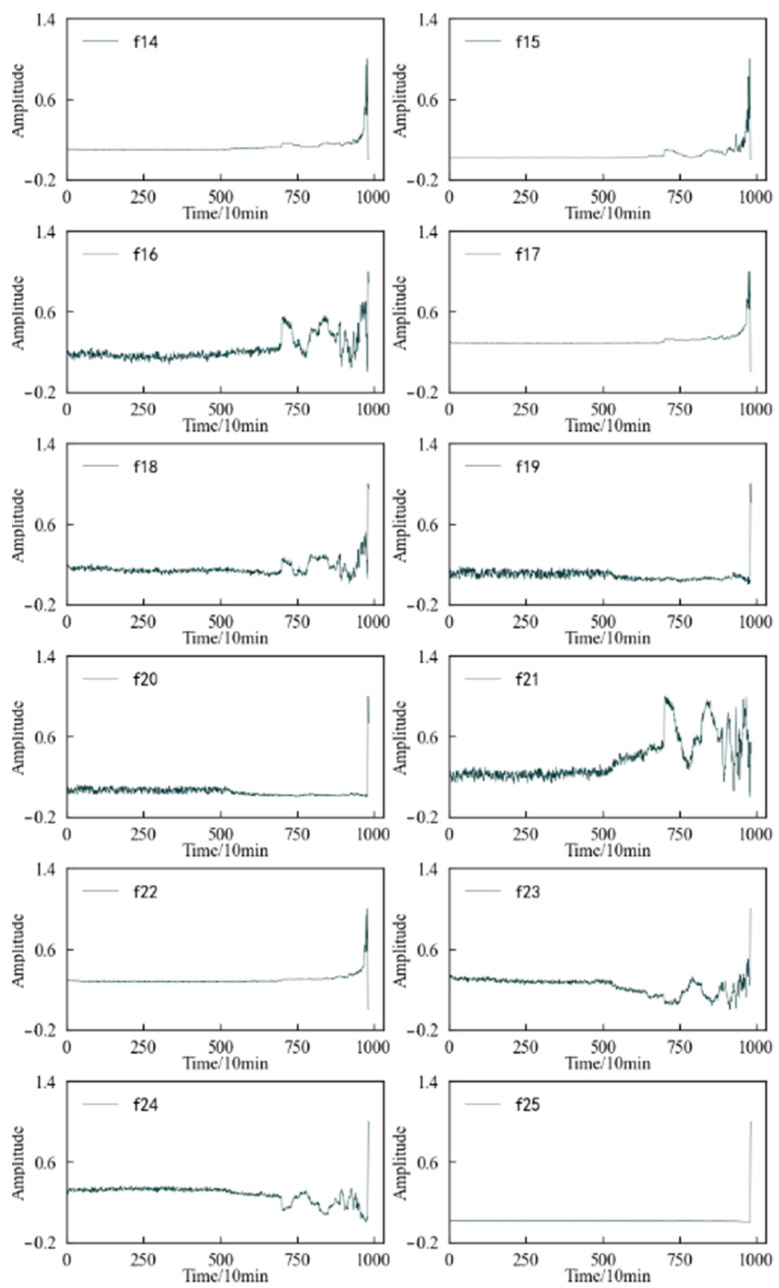
Variation trend in the frequency domain feature of 1#bearing.

**Figure 11:**
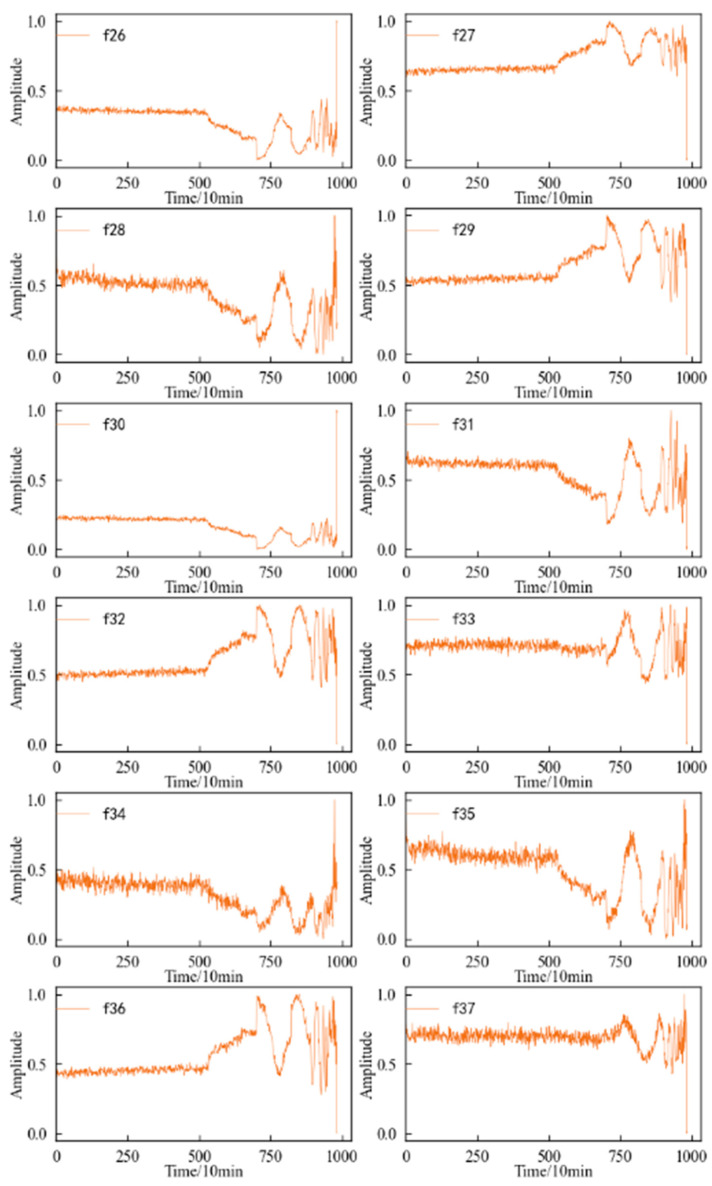
Variation trend in the time–frequency domain features of 1#bearing.

Combined with the experimental plan, an eigenvalue is calculated for the 20,480 sampling points collected in a single time, and each eigenindex sequence consists of 984 values. According to Table 1 and Table 2, the change trends in the time domain and frequency domain characteristics during the whole life cycle of the 1#bearing are shown in Figure 9 and Figure 10, respectively. Figure 11 shows the change trend in the time–frequency domain characteristics during the whole life cycle of the 1#bearing extracted using Equations (1) and (2). In order to visualize the change trend in each feature more conveniently, the features are normalized.

According to Figure 9, it can be determined that the time–domain features more intuitively reflect the performance degradation process of the 1#bearing than the original vibration signal, but not every time–domain feature describes this process well. The 13 time–domain features can be divided into the following four categories according to the changing trend: (1) The first type of feature, such as the mean characteristic, etc., is not sensitive to the slight and moderate faults of the 1#bearing, although the amplitude fluctuates from the beginning of the test to the end of the test. Until the 1#bearing fails completely, its amplitude changes significantly. (2) The second type of feature, such as the skewness and kurtosis characteristics, is relatively stable in the early stage, which corresponds well with the running state of the 1#bearing, but it is not sensitive enough to minor faults. After the 1#bearing is slightly damaged, the characteristics are not displayed in time and cannot show changes until the degree of failure is more serious. (3) The third type of feature, such as the peak index, margin index and pulse index, etc., fluctuates violently from the beginning and shows a certain change trend with the operation of the 1#bearing. But the fluctuation in this type of characteristics is too large, so that the change trend of the characteristics is not obvious enough to be submerged in it. (4) The changes in the fourth type of feature, such as the root mean square value, variance, and root square amplitude, are similar to those of the second type in the early stage, but the difference is that when the 1#bearing has a slight fault, the amplitude of the characteristics also changes correspondingly. And with a deepening of the failure degree, this change will be more obvious, which can better reflect the performance degradation trend of the bearing during its entire life.

According to Figure 10, it can be determined that the change process of the 12 frequency domain features over time is not the same. Except for the frequency domain feature 7, the other features change when the 1#bearing fails, which shows that the frequency domain features better reflect the performance degradation process of the 1#bearing. However, some frequency domain features, such as frequency domain features 1, 2, 5, and 6, decrease in amplitude after the 1#bearing failure and further decrease over time until the performance degrades sharply and their amplitude fluctuates drastically. 

According to Figure 11, it can be determined that most of the time–frequency domain features do not reflect the performance degradation process of the 1#bearing well, the trend is not obvious, and the fluctuation is large.

**Figure 12:**
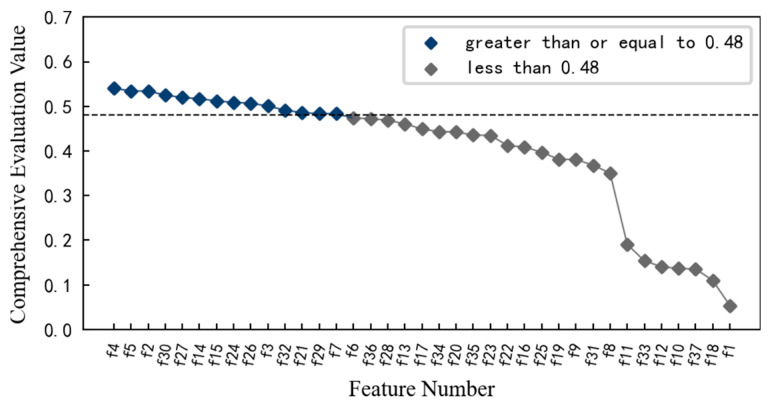
Comprehensive evaluation index of characteristics of 1#bearing.

Therefore, feature screening is carried out using the aforementioned method. At this time, the performance degradation of the 1#bearing is monitored, and α_1_ = 0.3, α_2_ = 0.3, and α_3_ = 0.4 during the calculation, and the result is shown in Figure 12. If the mean value of the comprehensive evaluation value of 37 features has 0.4013 as the threshold, then the comprehensive evaluation value of 25 features is greater than or equal to 0.4013. However, combined with the changing trends on the aforementioned features, it is found that some of these 25 features fluctuate greatly or are relatively gentle in the changing process, such as features *f*_16_ and *f*_22_. Therefore, considering the comprehensive evaluation value, the degradation process of each feature, and the dimension of the feature set, the features with a comprehensive evaluation value greater than or equal to 0.48 are reserved to form the optimal feature set, which includes 14 features such as features *f*_4_, *f*_5,_ and *f*_2_. Among these features, even including the feature *f*_7_ with the smallest comprehensive evaluation value, when the 1#bearing is running well, its amplitude fluctuation is small. Although it is not sensitive enough to minor damage, it can also be reflected after the obvious failure of the 1#bearing. Therefore, these 14 features can be used as the preferred feature set for performance degradation assessment of the 1#bearing.

### 3.2. Verification of the HI Construction Method

The KPCA method is used to reduce the dimensionality of the aforementioned 14 preferred features. First, the features of the low-dimensional space are mapped to the high-dimensional space. In this paper, the rbf radial basis function is used as the kernel function. The specific type is the Gaussian kernel function, and its expression is:

(14) $$k(x_{1},x_{2})=\exp(\frac{-\;{\left\|x_{1}-x_{2}\right\|}^{2}}{2\sigma })$$

where x_1_, x_2_ can be regarded as two feature sequences and σ is the kernel parameter.

**Figure 13:**
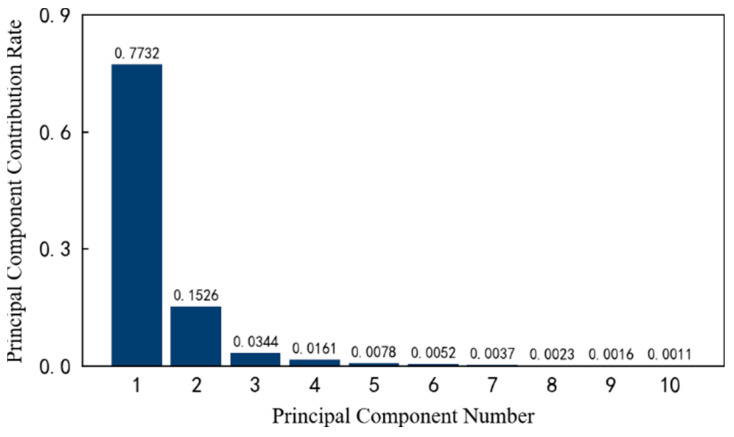
The contribution rate of the top 10 principal components of 1#bearing.

Secondly, the contribution rate of each principal component is calculated in a high-dimensional space. Taking the previous 10 principal components as an example, the respective contribution rates are shown in Figure 13. It can be seen that the first and second principal components account for 77.32% and 15.26% of the original data information, respectively. The larger the subsequent principal component number, the smaller the contribution rate, which gradually approach zero.

**Figure 14:**
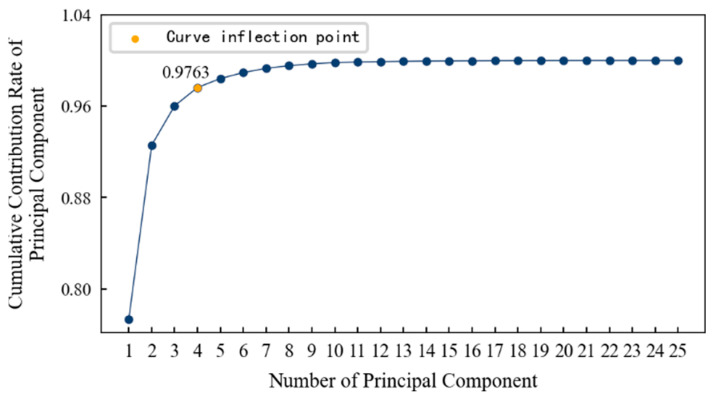
The cumulative contribution rate of the principal components of 1#bearing.

In order to determine the appropriate dimension, the cumulative contribution rate curve of the principal components is drawn, as shown in Figure 14. It can be seen that: (1) With an increase in the principal components, the cumulative contribution rate of principal components becomes smooth after a large increase and finally approaches 1 infinitely. (2) The inflection point on the curve is roughly at the fourth principal component. After that, even if the number of principal components continues to increase, the cumulative contribution rate will not increase significantly. Therefore, this paper selects the first four principal components as the features after dimension reduction, and their cumulative contribution rate reaches 97.63%. That is to say, the features after dimension reduction contain 97.63% of the information in the original data, and it is most appropriate that the dimension is reduced from 14 dimensions to 4 dimensions.

**Figure 15:**
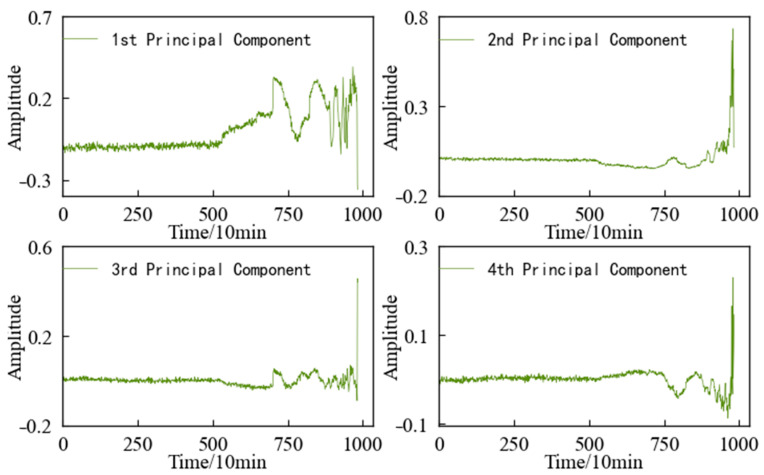
Variation trend in the top 4 principal components of 1#bearing.

The variation trends in the first four main elements of the 1#bearing are shown in Figure 15, which are used as the input value and target value of the CAE network for training, and the obtained encoded features are used as the HI of 1#bearing. For the convenience of comparison, the HI for the 1#bearing is constructed using an autoencoding (AE) network and a Gaussian mixture model (GMM), respectively. The HI obtained using the three different methods is shown in Figure 15 after denoising and smoothing.

**Figure 16:**
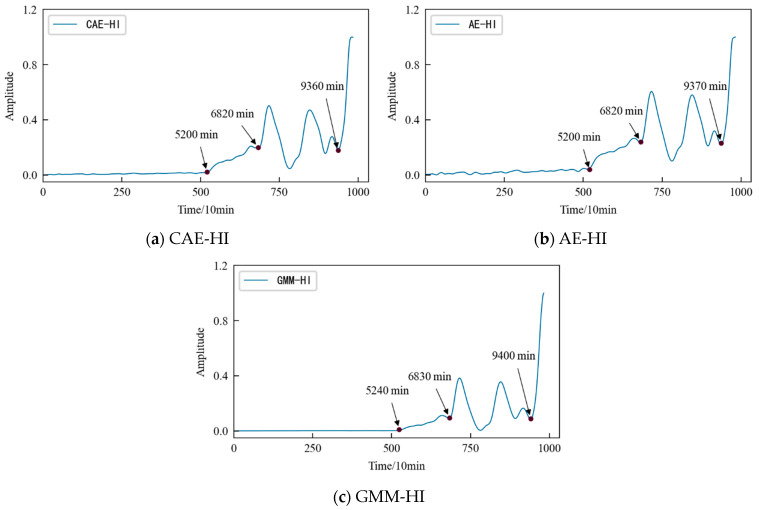
HI constructed using different methods of 1#bearing.

The comparative analysis shows that: (1) The HI constructed using the three different methods shows a consistent degradation trend, and the change process reflects the performance degradation process of the 1#bearing. (2) In the early stage of the test, since the running state of the 1#bearing is relatively stable, the amplitude of its HI should be relatively stable on the whole. Considering the actual operation situation, the amplitude of the HI slightly fluctuates more in line with reality. Figure 16 shows that the early stage of GMM-HI is too stable and has almost no fluctuation. The early stage of AE-HI fluctuates greatly, which is different from the actual situation, and the early stage of CAE-HI is in line with the expectation of overall stability and slight fluctuation. (3) As the test progresses, the 1#bearing begins to be damaged, and the amplitude of the HI should show an increasing trend. Figure 16 shows that GMM-HI began to increase significantly after the 5240th min, while AE-HI and CAE-HI began to increase after the 5200th min, which is 40 min ahead of GMM-HI. It is very important for the actual industrial production to be able to detect early damage to a monitored object in time. From this level of analysis, it is obvious that AE-HI and CAE-HI perform better. (4) When the 1#bearing fails, the amplitude of the HI should fluctuate greatly. Figure 15 shows that AE-HI and CAE-HI began to fluctuate greatly after the 6820th min, while GMM-HI was delayed by 10 min. (5) As the test continues, the 1#bearing fails completely, and the amplitude of the HI should change sharply. Figure 16 shows that CAE-HI shows a steep change at the 9360th min, while AE-HI and GMM-HI were delayed at the 9370th and 9400th min, respectively.

Therefore, whether from the qualitative analysis reflecting the early running state of the 1#bearing or from the quantitative analysis of the 1#bearing damage and failure time point, the results show that the CAE-HI constructed in this paper is superior to AE-HI and GMM-HI.

Further characteristic evaluation indicators, such as monotonicity and trend established using Equations (4) and (6), were used to evaluate the HI constructed using three different methods. The results are listed in Table 4. It can be seen that the CAE-HI constructed in this paper performs better than AE-HI and GMM-HI.

### 3.3. Verification of the Performance Degradation Prediction Model

Based on the TCN algorithm, the aforementioned direct multi-step prediction is used to predict the performance degradation trend of 1#bearing. By comparing the results with the prediction models constructed using the long short-term memory (LSTM) network and the gated recurrent unit (GRU) algorithm, the superiority of the performance degradation prediction method proposed in this paper is verified. 

**Figure 17:**
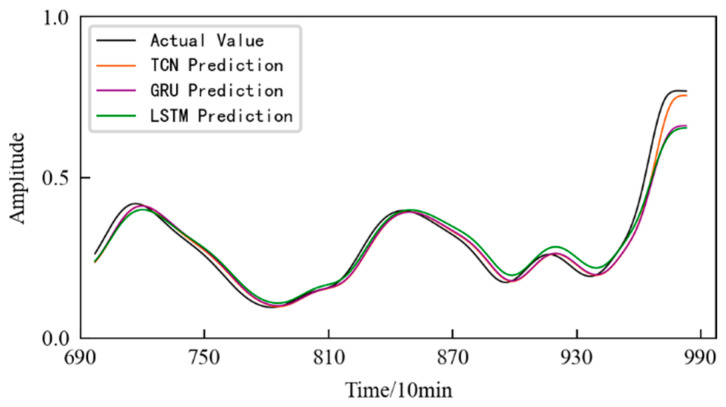
Performance degradation prediction for the 1#bearing based on different methods.

The HI of the 1#bearing has a total of 984 data files, which are divided into a training set and a test set according to the ratio of 7:3, that is, the first 689 data files and the remaining 295 data files, respectively, form the training set and the test set for the prediction model using spatial phase reconstruction technology. The time step for each input was set to 8 and the prediction step was set to 3. The size of the convolution kernel in the model was set to 3, the number of convolution layers was set to 4, and the expansion coefficient was set to 2. The number of iterations for training the model was 150, and the input batch size in each training was 10. The prediction results based on the TCN model are shown in Figure 17, which also shows the prediction results obtained using the LSTM and GRU models. It should be noted that in order to prevent the model from overfitting, the number of hidden layers for training the LSTM and GRU models was set to 1, the maximum number of iterations was 100, and the dropout was 0.3.

According to Figure 17, it can be seen that: (1) The prediction models established using the three different methods make accurate performance degradation predictions for the 1#bearing, and the difference between the predicted values of three different methods at certain time points is very small or even the same. (2) In the failure stage of the 1#bearing, the deviation between the predicted value and the actual value of the two methods using the LSTM algorithm and the GRU algorithm is large.

Further, combined with the evaluation indicators established using Equations (12) and (13), the prediction models constructed using the three different methods were evaluated, and the results are shown in Table 5. Compared with the other two models, the performance degradation trend prediction model constructed in this paper using TCN has better prediction performance and higher prediction accuracy.

## 4. Application

The feasibility of the performance degradation trend prediction method based on multi-domain features and TCN proposed in this paper was verified using bearing public data. In this section, the model is applied to predict the performance degradation trend of a rolling contact fatigue specimen to further verify the effectiveness of the method.

### 4.1. Rolling Contact Fatigue Test

**Figure 18:**
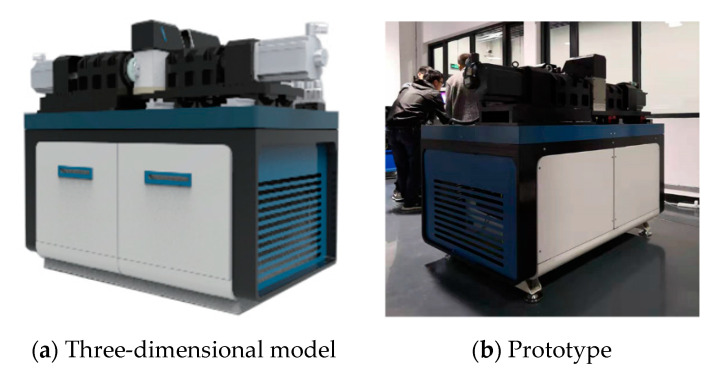
The structure of RCF-A machine.

According to the “Rolling Contact Fatigue Test Method for Metal Materials” (YB/T 5345.2014) [41], rolling contact fatigue testing is the only way to obtain the contact fatigue properties of materials. The research group successfully developed the RCF-A machine, as shown in Figure 18, which solved the technical problems of similar test machines, such as discontinuous data collection and difficulty in accurately obtaining fatigue strength.

**Figure 19:**
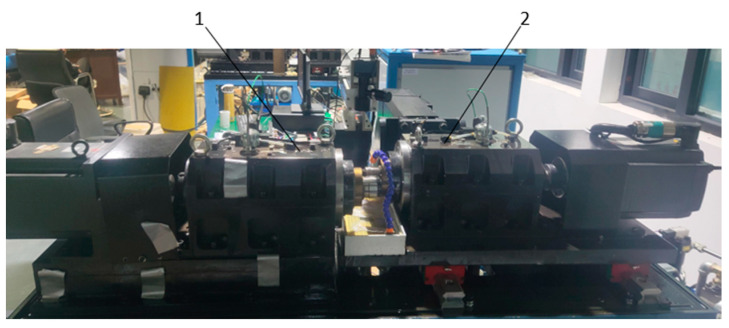
Schematic diagram showing the shaft box of an RCF-A machine. 1: Accompanying axle box and 2: spindle box.

**Figure 20:**
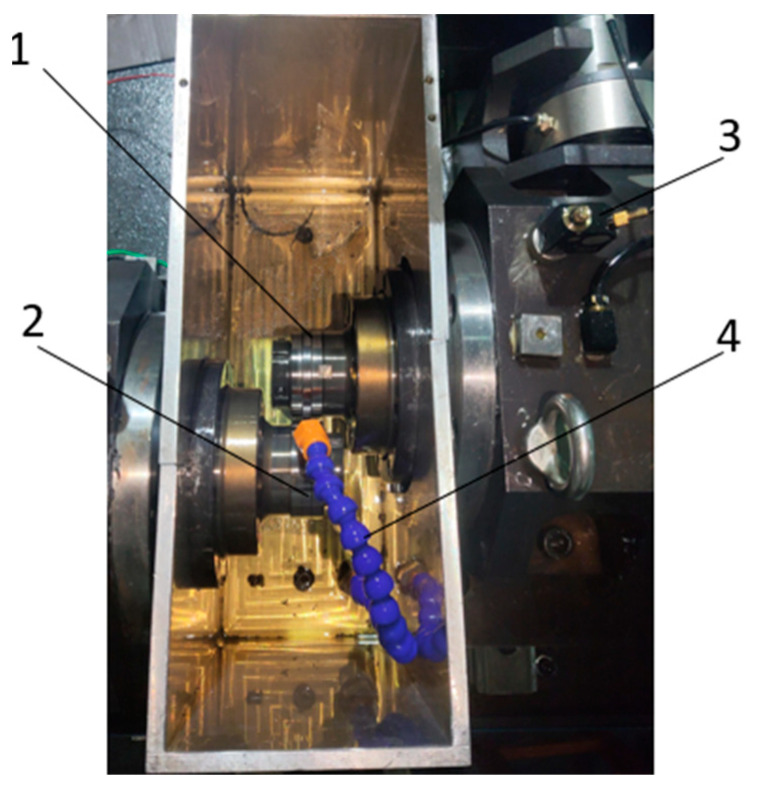
The installation position of the accelerometer. 1: Specimen; 2: accompanying specimen; 3: acceleration sensor; and 4: fuel injection pipe.

**Figure 21:**
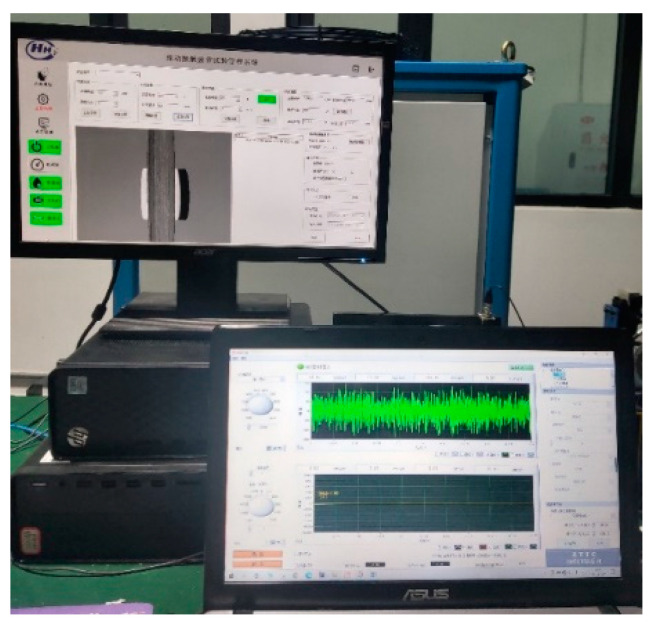
The control interface of the RCF-A machine.

A test was carried out using an RCF-A machine, as shown in Figure 19. The 1A307E accelerometer is installed on the headstock box with magnetic attraction (as shown in Figure 20), and the EM9118B-6/ICP data acquisition card is used to collect vibration signals. The working conditions were set as shown in Table 6, and the control interface of the RCF-A machine is shown in Figure 21. 

**Figure 22:**
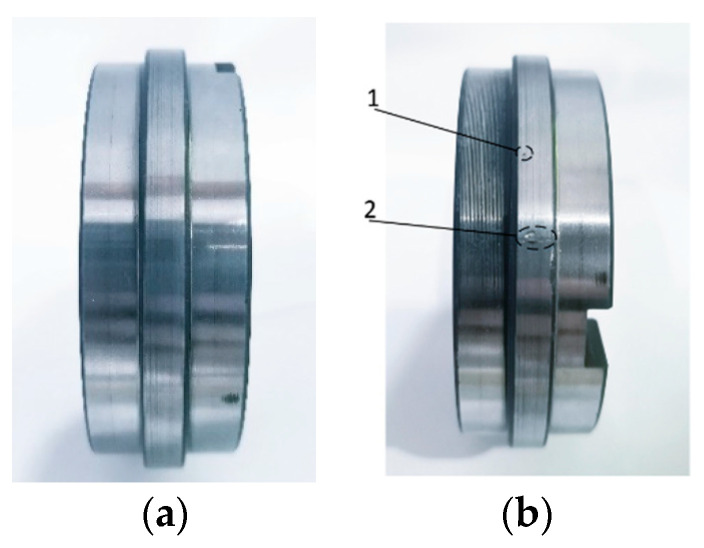
Comparison of specimen before and after the test. (**a**) Before the test and (**b**) after the test. 1: Pitting and 2: crack spalling.

**Figure 23:**
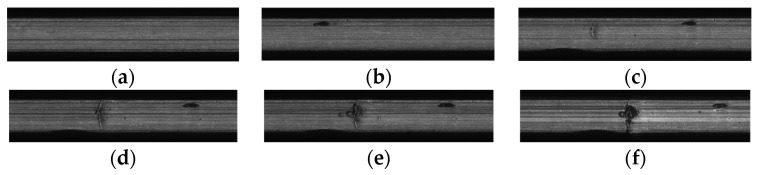
Fatigue damage and evolution process of the specimen surface. (**a**) Normal status; (**b**) pitting; (**c**) cracks appear; (**d**) crack propagation; (**e**) further crack growth; and (**f**) flaking occurs.

Finally, when the test reached 1632 min, the contact fatigue failure of the specimen occurred. Figure 22 shows the good state and failure state of the specimen before and after the test. It can be seen that due to rolling contact fatigue, pitting corrosion and crack-induced spalling occurred at 1 and 2 in Figure 22b, respectively. Although not easily visible to the naked eye, with the help of the image acquisition system of the testing machine, the time of fatigue failure was accurately captured. Figure 23 shows the acquired image information for the contact surface of the specimen at key time points, which truly records the evolution process in the contact surface of the sample from the initiation of fatigue damage to the appearance of cracks and then to spalling.

**Figure 24:**
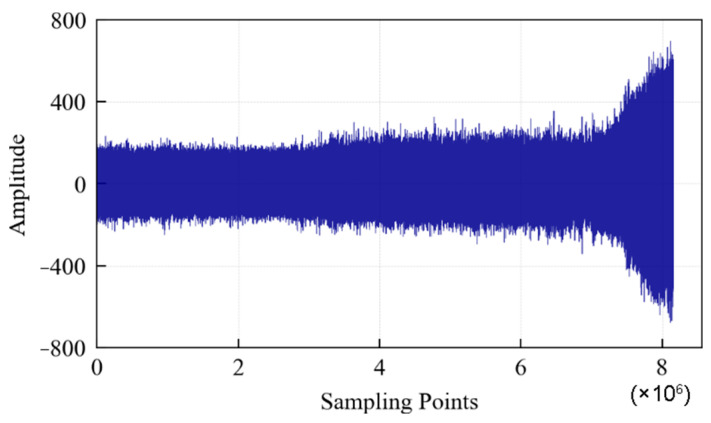
Vibration signal during the whole life cycle of the specimen.

According to the above-mentioned test process and data collection method, if the data collected every 1 s is a data file and each file contains 10,000 sampling points, then a total of 816 data files are obtained until the specimen fails. The vibration signal during the whole life cycle of the specimen is shown in Figure 24, which contains 8.16 × 10^6^ sampling points. It can be seen that: (1) From the beginning of the test to about 3 × 10^6^ sampling points, the value of the specimen vibration signal does not fluctuate much, indicating that the specimen is in a relatively stable state. (2) From 3 × 10^6^ to 7 × 10^6^ sampling points, the signal amplitude increases significantly. Fatigue pitting damage should have occurred in the specimen. (3) After 7 × 10^6^ sampling points, the signal amplitude continued to increase, indicating that cracks appeared on the surface of the specimen, and the crack area expanded rapidly. Finally, spalling was formed, and the specimen failed completely.

### 4.2. Feature Screening

**Figure 25:**
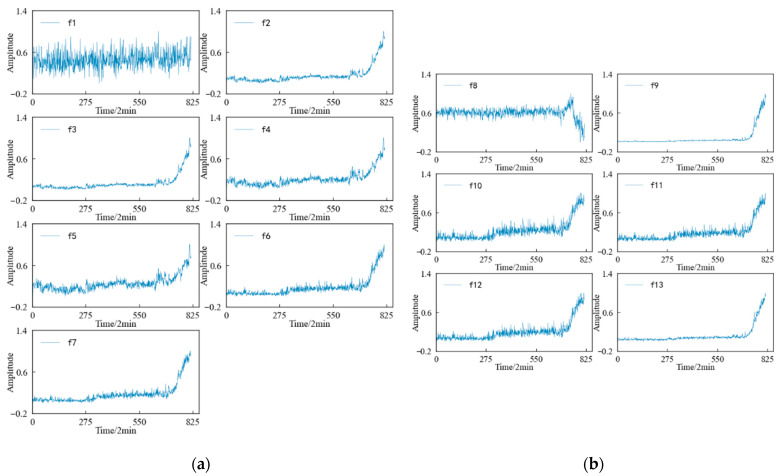
Variation trend in time domain feature of specimen. (**a**) Dimensional feature. (**b**) Dimensionless features.

A feature value is calculated for the 1 × 10^4^ data points obtained at each sampling time, and each feature sequence has a total of 816 values. The variation trend in the time domain characteristics of the specimen during the whole life cycle is shown in Figure 25. It can be seen that: (1) In the dimensioned time domain feature, the mean value feature is not sensitive to the contact fatigue of the specimen, and the amplitude changes in the other features can better reflect the state change in the specimen compared with the original vibration. The signal is more timely and clear. (2) Among the dimensionless time domain features, in addition to the skewness index feature, te other features can also better reflect the change process in the specimen from normal to failure.

**Figure 26:**
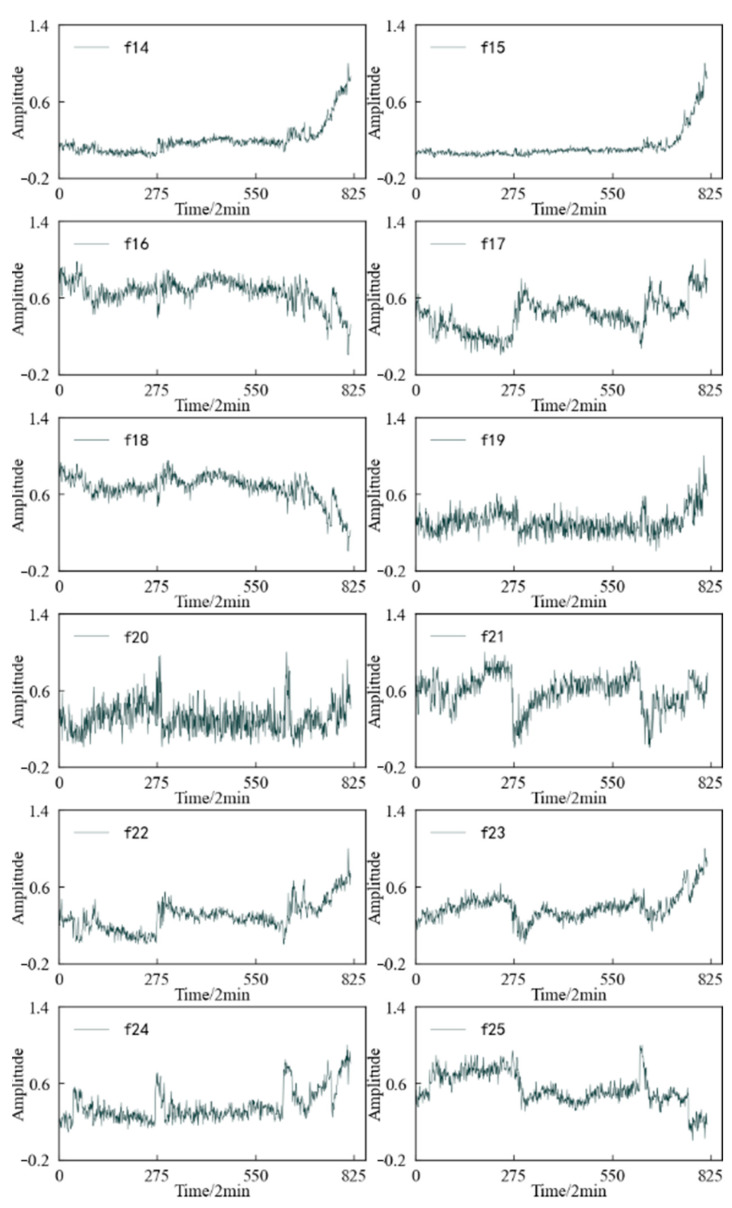
Variation trend in the frequency domain feature of specimen.

Figure 26 shows the variation trend in the frequency domain characteristics of the specimen during the whole life cycle. It can be found that in terms of reflecting the operating state of the specimen, the overall performance of the frequency domain features is not as good as the time domain features. Except for the amplitude mean and amplitude variance, the other frequency domain features have large fluctuations during the whole life cycle. Although there is a certain trend in the performance degradation process of the specimen, this trend is not stable, and even jumps at some time points.

**Figure 27:**
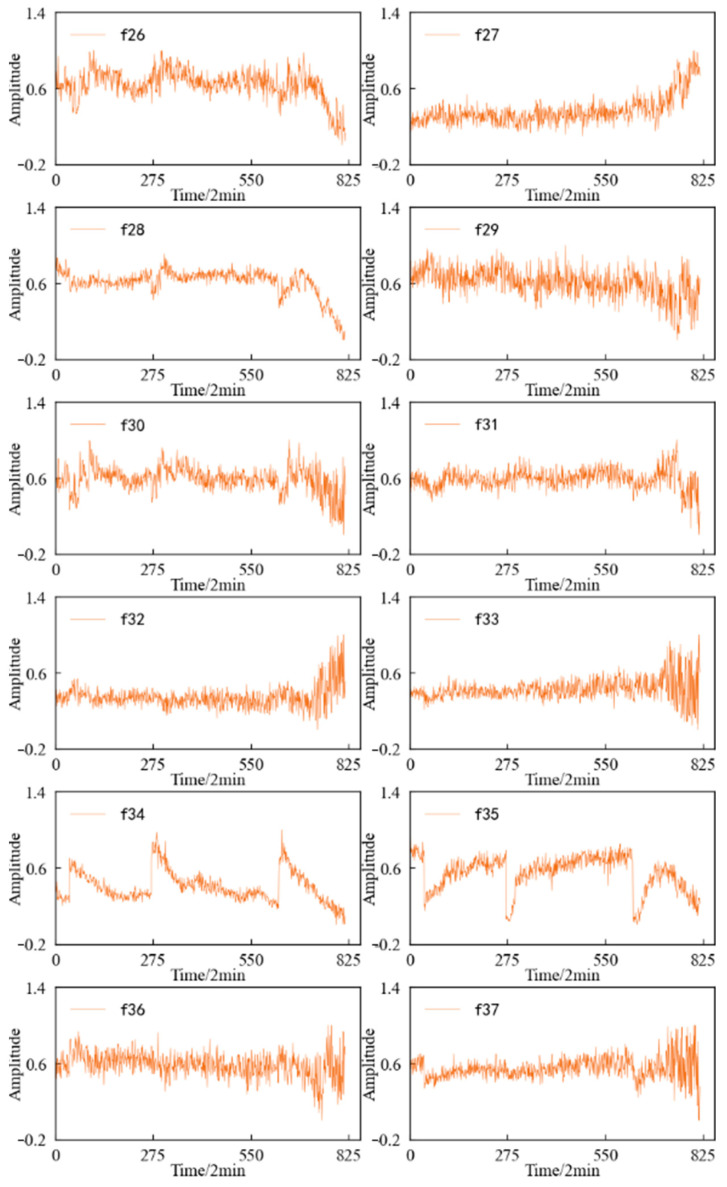
Variation trend in the time–frequency domain features of specimen.

Three-layer wavelet packet decomposition is performed on the vibration signal of the specimen, and the obtained energy changes in each frequency band in the second layer and the third layer are shown in Figure 27. It can be seen that most of the time–frequency domain features do not reflect the performance degradation process of the specimen well, the trend is not obvious, and the fluctuation is large.

**Figure 28:**
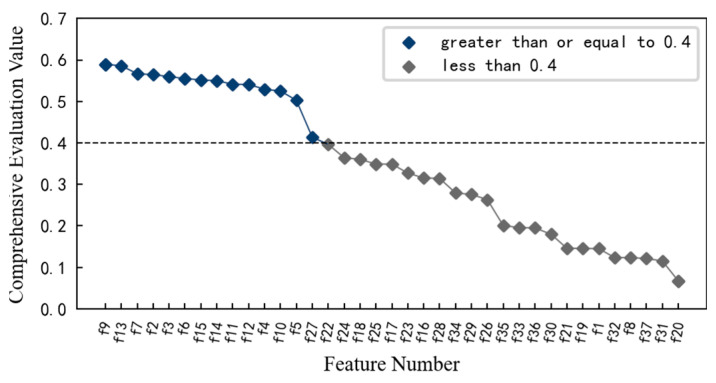
Comprehensive evaluation index value of each feature of specimen.

Similarly, in order to obtain the features that are sensitive to the contact fatigue degradation process of the specimen, the monotonicity, correlation mean and trend of 37 multi-domain features are calculated using Equations (4)–(6). On this basis, the comprehensive evaluation index for each feature is calculated using Equation (3), and the result is shown in Figure 28. Considering the comprehensive evaluation value and degradation process of each feature, and considering the dimension of the feature set, the features with comprehensive evaluation value greater than or equal to 0.4 are reserved to form the optimal feature set, which includes 14 features such as feature *f*_9_, feature *f*_13_, and feature *f*_7_. These preferred features better reflect the performance degradation process of the specimen.

### 4.3. Feature Dimensionality Reduction

The rolling contact fatigue performance of the specimen is degraded, and its vibration signal also has non-stationary and nonlinear characteristics. Dimensionality reduction of the aforementioned 14 preferred features was carried out using KPCA method.

**Figure 29:**
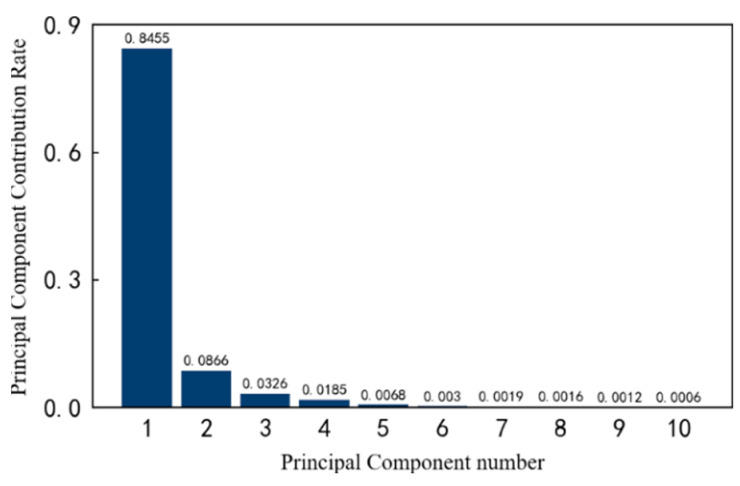
The contribution rate of the top 10 principal components of specimen.

**Figure 30:**
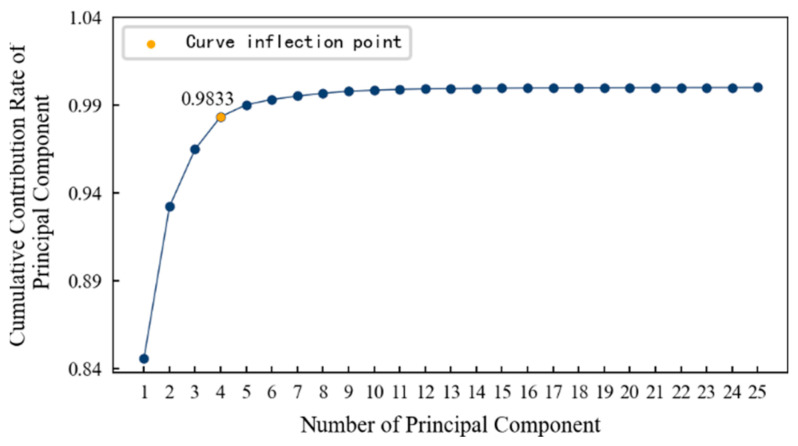
The cumulative contribution rate of the principal components of specimen.

When mapping the features of the low-dimensional space to the high-dimensional space, the Gaussian kernel function, shown in Equation (14), is used, and the respective contribution rates of the first 10 principal components are calculated, as shown in Figure 29. The plotted pivotal cumulative contribution rate curve is shown in Figure 30. It can be seen that: (1) As the number of principal components increases, the cumulative contribution rate of the principal components also increases, and more information is contained. (2) The inflection point on the curve is roughly at the fourth principal component. At this time, even if the number of principal components continues to increase, the cumulative contribution rate will not increase significantly. Therefore, it is most appropriate to reduce the dimensions from 14 to 4.

**Figure 31:**
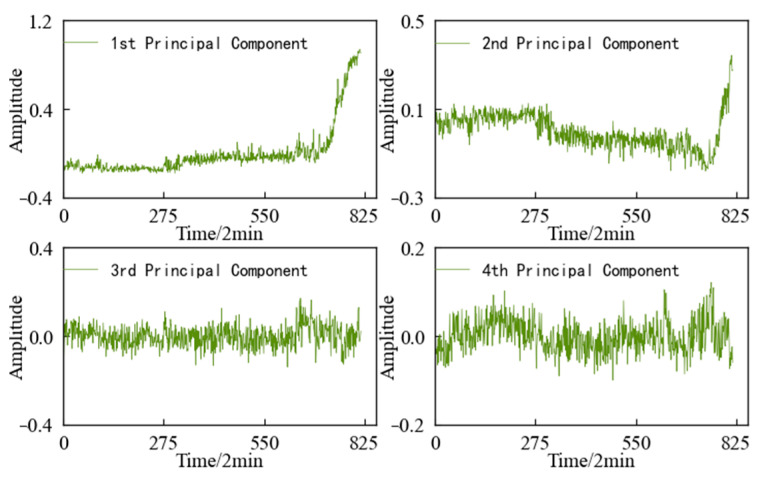
Variation trend in the top 4 principal components of specimen.

The variation trends in the first four principal components of the specimen are shown in Figure 31. Using feature dimensionality reduction, the optimal feature set of the specimen is reduced from 14 dimensions to 4 dimensions. After dimensionality reduction, the feature set contains 98.33% of the information in the optimal feature set, achieving the purpose of reducing the dimension without losing a lot of information.

### 4.4. Construction of the HI

**Figure 32:**
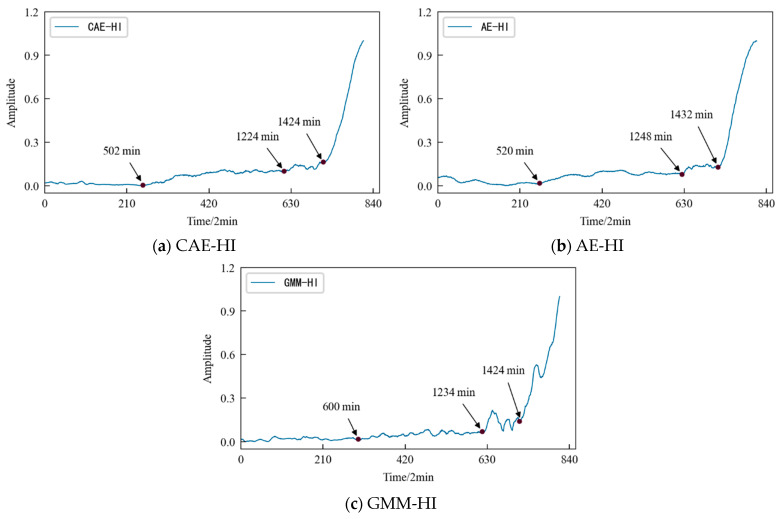
HI constructed using different methods of specimen.

The dimensionality-reduced features are used as the input value and target value for the CAE network for training, and the obtained encoded features are used as the HI for the specimen. Similarly, the AE network and the GMM are used to construct the HI for the specimen, respectively, for comparison. The HI obtained using the three different methods were denoised and smoothed, as shown in Figure 32.

The comparative analysis shows that: (1) The HI constructed using the three different methods show a consistent degradation trend, and the change process reflects the performance degradation process of contact fatigue in the specimen, which is consistent with the process of rolling contact fatigue in the specimen in Figure 23. (2) In the early stage of the test, due to the relatively stable running state of the specimen, the amplitudes of the HI were generally stable, but slightly fluctuated due to the influence of the test environmental conditions. (3) With the progress of the test, the specimen became damaged, and the amplitude of the HI showed an increasing trend. Figure 32 shows that AE-HI and GMM-HI began to increase significantly after 520 min and 600 min, respectively, while CAE-HI began to show an increasing trend after 502 min, i.e., compared with AE-HI and GMM-HI, it was advanced by 18 min and 98 min, respectively. Therefore, the HI construction method proposed in this paper has more advantages. (4) When the specimen has serious fatigue damage such as a crack, the amplitude of the HI should fluctuate greatly. Figure 32 shows that CAE-HI began to fluctuate greatly after 1224 min, while the large fluctuations in AE-HI and GMM-HI were delayed by 24 min and 10 min, respectively. (5) With the continuous progress of the test, the fatigue failure of the specimen occurred, and the amplitude of the HI changes sharply. Figure 32 shows that CAE-HI and GMM-HI had a steep change at 1424 min, while the abrupt change in AE-HI appeared at 1432 min, and there was a lag.

In a word, whether from the qualitative analysis reflecting the early running state of the specimen or from the quantitative analysis of the time point of the fatigue damage and fatigue failure of the specimen, the results show that the CAE-HI constructed in this paper is superior to AE-HI and GMM-HI. Therefore, the method of constructing HI based on CAE network is reasonable and effective, and this method has obvious advantages compared with the AE network and GMM, two commonly used methods of constructing an HI.

### 4.5. Performance Degradation Prediction of Rolling Contact Fatigue

Based on the TCN algorithm, the direct multi-step prediction is also used for performance degradation prediction of the rolling contact fatigue specimen. After comparing the results with the prediction models using the LSTM algorithm and the GRU algorithm, the superiority of the proposed performance degradation trend prediction method is further verified.

The HI of the specimen has a total of 816 data files, which are divided into a training set and a test set according to the ratio of 7:3, that is, the first 571 data files and the remaining 245 data files, respectively, form the training set and the test set, respectively, for the prediction model using spatial phase reconstruction technology. The other parameters are the same as that of the performance degradation prediction of the 1#bearing.

**Figure 33:**
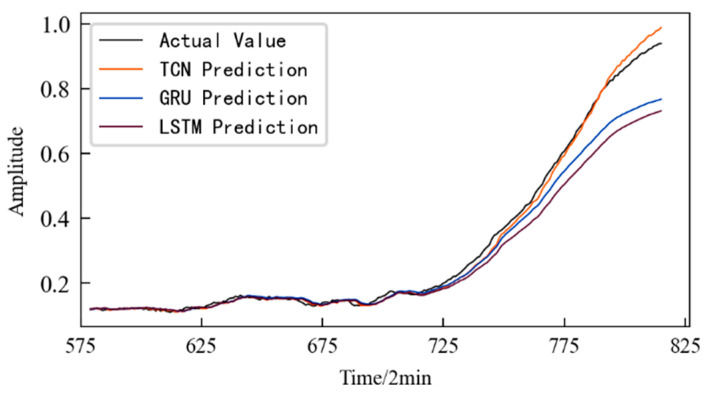
Prediction results of the different prediction models.

The prediction results obtained using the TCN model are shown in Figure 33, and the prediction starts from the 580th sampling time, that is, the 1160th min. In order to illustrate the superiority of the method, the prediction results obtained using two commonly used time series data prediction models, LSTM network and GRU, are also shown in Figure 33. The evaluation indicators for three different prediction models are listed in Table 7. It can be seen that the prediction model based on the TCN predicts the performance degradation of the rolling contact fatigue specimen well. Moreover, it has the best fit with the actual performance degradation curve, and the prediction accuracy is higher. At the same time, the model evaluation index is also the best among the three models.

## 5. Discussion

**Figure 34:**
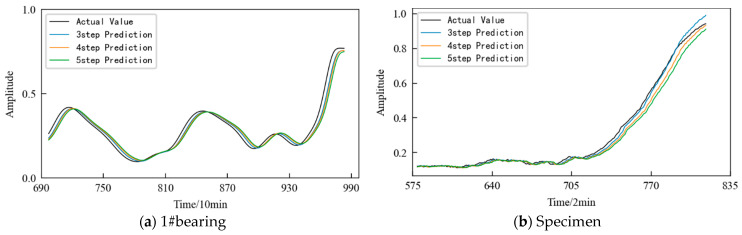
Prediction results obtained using TCN under different prediction step sizes.

In practical applications, the number of predicted steps can be appropriately increased according to demand. Figure 34 shows the prediction results obtained using the TCN model in the prediction time period when the prediction steps are 3, 4, and 5, respectively. It can be seen that: (1) In the early stage of prediction, the difference between the predicted values under different prediction step sizes is small and close to the actual value. (2) With the passage of time, the variation range of the HI increases, and the predicted value begins to deviate from the actual value. (3) In the later stage of prediction, the prediction error increases. The larger the prediction step size, the larger the error.

Further, the evaluation indicators RMSE and MAE are used to quantitatively evaluate the prediction effect of the model. The evaluation index values for the models under the aforementioned three prediction steps are listed in Table 8. It can be seen that under the same other conditions, with an increase in the prediction step size each time, the error in the prediction results increases. Therefore, when predicting the performance degradation trend of the monitored object, the prediction step size can be reasonably selected according to the actual demand under the premise of satisfying the prediction accuracy.

## 6. Conclusions

In this paper, a performance degradation prediction method based on multi-domain features and TCN is proposed and implemented, comprehensively using theories and methods such as multi-feature fusion, feature space transformation, and machine learning.

(1) In terms of HI construction, in view of the information loss problem faced by the construction of one-dimensional HI using multi-domain feature extraction, on the basis of multi-domain feature extraction, a method using KPCA to reduce feature dimensionality and CAE network to construct performance degradation HI is proposed. The results show that KPCA can reduce the dimension of the feature set and retain more than 97% of the information in the original data. Furthermore, the change process of the HI constructed using the CAE network truly reflects the performance degradation process of the monitored object. Compared with the AE network and GMM, this method has obvious advantages.

(2) Regarding prediction model construction, in view of the shortcomings of the commonly used prediction model based on recurrent neural networks, such as low parallel computation and small receptive field of input data, a performance degradation trend prediction method based on multi-domain features and TCN is proposed. The results show that the prediction model constructed using the TCN algorithm can accurately predict the performance degradation trend of the monitored object, and the constructed prediction model has better performance and higher accuracy than the prediction models using the LSTM network and GRU.

The verification results for the bearing public data and the application results for the rolling contact fatigue show that the performance degradation prediction method based on multi-domain features and TCN proposed in this paper is feasible and effective. This method has general significance and can be extended to the performance degradation prediction of other mechanical equipment/components.

Currently, big data technology and machine learning are booming. In future research, an intelligent extraction method for health indexes should be considered to directly obtain health indexes from feature sets or even from the raw vibration signal without influencing the calculation speed. At the same time, it is necessary to simplify the implementation process of forecasting methods, improve computing efficiency, and meet real-time forecasting needs.

## Figures and Tables

**Table 1:** Time domain feature parameters.

No.	Feature	Calculation Formula
Dimensional Time Domain Features		
*f*_1_	mean	$\overset{-}{X}=\frac{1}{N}\sum_{i=1}^{N}x_{i}$
*f*_2_	rms value	$X_{rms}=\sqrt{\frac{1}{N}\sum_{i=1}^{N}x_{i}^{2}}$
*f*_3_	variance	$X_{\mathrm{var}}=\frac{1}{N-1}\sum_{i=1}^{N}{\left(x_{i}-\bar{X}\right)}^{2}$
*f*_4_	absolute mean	${\bar{X}}_{abs}=\frac{1}{N}\sum_{i=1}^{N}\left|x_{i}\right|$
*f*_5_	root amplitude	$X_{sra}={\left(\frac{1}{N}\sum_{i=1}^{N}\sqrt{\left|x_{i}\right|}\right)}^{2}$
*f*_6_	peak	$X_{p}=\underset{1\leq i\leq N}{\max}\left(\left|x_{i}\right|\right)$
*f*_7_	peak to peak	$X_{p-p}=\max\left(x_{i}\right)-\min\left(x_{i}\right)$
Dimensionless time domain features		
*f*_8_	skewness index	$X_{skew}=\frac{1}{N}\sum_{i=1}^{N}{\left(x_{i}-\bar{X}\right)}^{3}/X_{std}{}^{3}$
*f*_9_	kurtosis index	$X_{kur}=\frac{1}{N}\sum_{i=1}^{N}{\left(x_{i}-\bar{X}\right)}^{4}/X_{std}{}^{4}$
*f*_10_	peak indicator	$X_{c-f}=X_{p}/X_{rms}$
*f*_11_	margin indicator	$X_{cl-f}=X_{p}/Xsra$
*f*_12_	impulse indicator	$X_{i-f}=X_{p}/{\bar{X}}_{abs}$
*f*_13_	waveform indicator	$X_{s-f}=X_{rms}/{\bar{X}}_{abs}$

**Table 2:** Frequency domain feature parameters.

No.	Feature	Calculation Formula
*f*_14_	frequency amplitude mean	$F_{1}=\frac{1}{K}\sum_{k=1}^{K}s\left(k\right)$
*f*_15_	frequency amplitude variance	$F_{2}=\frac{1}{K-1}\sum_{k=1}^{K}{\left(s\left(k\right)-F_{1}\right)}^{2}$
*f*_16_	first-order center of gravity	$F_{3}=\sum_{k=1}^{K}f_{k}s\left(k\right)/\sum_{k=1}^{K}s\left(k\right)$
*f*_17_	second-order center of gravity	$F_{4}=\sqrt{\frac{1}{K}\sum_{k=1}^{K}{\left(f_{k}-F_{3}\right)}^{2}s\left(k\right)}$
*f*_18_	rms frequency	$F_{5}=\sqrt{\sum_{k=1}^{K}f_{k}{}^{2}s\left(k\right)/\sum_{k=1}^{K}s\left(k\right)}$
*f*_19_	frequency domain features 1	$F_{6}=\sum_{k=1}^{K}{\left(s\left(k\right)-F_{1}\right)}^{3}/\left[K{\left(\sqrt{F_{2}}\right)}^{3}\right]$
*f*_20_	frequency domain features 2	$F_{7}=\sum_{k=1}^{K}{\left(s\left(k\right)-F_{1}\right)}^{4}/\left(KF_{2}{}^{2}\right)$
*f*_21_	frequency domain features 3	$F_{8}=\sum_{k=1}^{K}f_{k}{}^{2}s\left(k\right)/\sqrt{\sum_{k=1}^{K}s\left(k\right)\sum_{k=1}^{K}f_{k}{}^{4}s\left(k\right)}$
*f*_22_	frequency domain features 4	*F*_9_ = *F*_4_/*F*_3_
*f*_23_	frequency domain features 5	$F_{10}=\sqrt{\sum_{k=1}^{K}f_{k}{}^{4}s\left(k\right)/\sum_{k=1}^{K}f_{k}{}^{2}s\left(k\right)}$
*f*_24_	frequency domain features 6	$F_{11}=\sum_{k=1}^{K}{\left(f_{k}-F_{3}\right)}^{3}s\left(k\right)/\left(KF_{4}{}^{3}\right)$
*f*_25_	frequency domain features 7	$F_{12}=\sum_{k=1}^{K}{\left(f_{k}-F_{3}\right)}^{4}s\left(k\right)/\left(KF_{4}{}^{4}\right)$

**Table 3:** The network architecture and parameters of CAE.

Network Layer	Dimensions Entered	Size of Convolution Kernel	Number of Convolution Kernels	Dimensions of the Output
Convolutional layer 1	4 × 1	2 × 1	5	3 × 5
Convolutional layer 2	3 × 5	2 × 1	5	2 × 3
Convolutional layer 3	2 × 3	2 × 1	1	1 × 1
Transpose convolutional layer 1	1 × 1	2 × 1	4	2 × 4
Transpose convolutional layer 2	2 × 4	2 × 1	4	3 × 4
Transpose convolutional layer 3	3 × 4	2 × 1	2	4 × 1

**Table 4:** Performance of the HI constructed using different methods.

Evaluation Indicator	CAE-HI	AE-HI	GMM-HI
Monotonicity	0.2513	0.2411	0.1801
Trend	0.9462	0.9430	0.9454

**Table 5:** Evaluation metrics for the different prediction models.

Evaluation Indicator	Predictive Model		
	TCN	LSTM	GRU
RMSE	0.0257	0.0385	0.0366
MAE	0.0187	0.0264	0.0234

**Table 6:** Working conditions.

Specimen Material	Rotational Speed (r/min)		Slip Rate	Radial Load (N)	Sampling		
	Main Shaft (Specimen)	Accompanying Shaft (Accompanying Specimen)			Frequency (kHz)	Single Sample Duration (s)	Sampling Interval (min)
40Cr	1000	1100	10%	2071	10	1	2

**Table 7:** Evaluation index for the different prediction models.

Evaluation Indicators	Predictive Model		
	TCN	GRU	LSTM
RMSE	0.0146	0.0555	0.0744
MAE	0.0105	0.0308	0.0423

**Table 8:** Evaluation index for TCN model under different prediction step size.

Evaluation Indicator	1#Bearing			Specimen		
	Prediction Step Size					
	3	4	5	3	4	5
RMSE	0.0257	0.0333	0.0418	0.0146	0.0259	0.0393
MAE	0.0187	0.0243	0.0305	0.0105	0.0164	0.0270

## Data Availability

Not applicable.
